# To develop with or without the prion protein

**DOI:** 10.3389/fcell.2014.00058

**Published:** 2014-10-13

**Authors:** Sophie Halliez, Bruno Passet, Séverine Martin-Lannerée, Julia Hernandez-Rapp, Hubert Laude, Sophie Mouillet-Richard, Jean-Luc Vilotte, Vincent Béringue

**Affiliations:** ^1^Institut National de la Recherche Agronomique, U892 Virologie et Immunologie MoléculairesJouy-en-Josas, France; ^2^Institut National de la Recherche Agronomique, UMR1313 Génétique Animale et Biologie IntégrativeJouy-en-Josas, France; ^3^Institut National de la Santé et de la Recherche Médicale, UMR-S1124Paris, France; ^4^Université Paris Descartes, Sorbonne Paris Cité, UMR-S1124Paris, France

**Keywords:** prion protein, development, neural development, stem cells, cell adhesion, extra-cellular matrix, cytoskeleton

## Abstract

The deletion of the cellular form of the prion protein (PrP^C^) in mouse, goat, and cattle has no drastic phenotypic consequence. This stands in apparent contradiction with PrP^C^ quasi-ubiquitous expression and conserved primary and tertiary structures in mammals, and its pivotal role in neurodegenerative diseases such as prion and Alzheimer's diseases. In zebrafish embryos, depletion of PrP ortholog leads to a severe loss-of-function phenotype. This raises the question of a potential role of PrP^C^ in the development of all vertebrates. This view is further supported by the early expression of the PrP^C^ encoding gene (*Prnp*) in many tissues of the mouse embryo, the transient disruption of a broad number of cellular pathways in early *Prnp^−/−^* mouse embryos, and a growing body of evidence for PrP^C^ involvement in the regulation of cell proliferation and differentiation in various types of mammalian stem cells and progenitors. Finally, several studies in both zebrafish embryos and in mammalian cells and tissues in formation support a role for PrP^C^ in cell adhesion, extra-cellular matrix interactions and cytoskeleton. In this review, we summarize and compare the different models used to decipher PrP^C^ functions at early developmental stages during embryo- and organo-genesis and discuss their relevance.

## Introduction

Prion diseases are a group of fatal and transmissible neurodegenerative diseases affecting a broad range of mammals including humans. The causative agent (the prion) is primarily composed of abnormally folded and aggregated forms of a host-encoded protein, the cellular prion protein (PrP^C^). PrP^C^ is a glycosyl-phosphatidyl-inositol anchored cell surface sialoglycoprotein associated with lipid rafts (Taylor et al., 2009). PrP primary sequence is highly conserved among mammals (Wopfner et al., 1999) and PrP putative functional domains are structurally conserved between mammals, avians, and fish (Wopfner et al., 1999; Rivera-Milla et al., 2006). PrP^C^ is widely expressed in nearly all the organism, albeit at highest levels in the adult nervous system (Bendheim et al., 1992). Together, these data lend support for an essential role of PrP^C^ in mammals and possibly in vertebrates in general. However, the production of mice, goat, and cattle lacking PrP did not lead to any obvious phenotype (Bueler et al., 1992; Manson et al., 1994a; Richt et al., 2007; Yu et al., 2009) except, for mice, a resistance to experimental prion diseases (Bueler et al., 1993; Prusiner et al., 1993; Manson et al., 1994b; Mallucci et al., 2003). Additionally, goats, naturally devoid of PrP^C^ due to a nonsense mutation, do not seem to present any abnormal phenotype (Benestad et al., 2012). Subtle behavioral and oxidative stress-related alterations have been then reported in adult mice devoid of PrP^C^ (see Table 1) (Tobler et al., 1996; Wong et al., 2001; Roucou et al., 2004; Meotti et al., 2007; Sanchez-Alavez et al., 2008; Le Pichon et al., 2009; Gadotti et al., 2012) although some of them are debated (Steele et al., 2007) and some may be related to *Prnp*-flanking genes rather than to PrP^C^ absence itself (Nuvolone et al., 2013). However, none of them seems, at first glance, so important to justify the conserved structure and broad expression of PrP^C^. The physiological role of PrP^C^ still remains highly uncertain despite more than two decades of research and numerous proposed functions (Nicolas et al., 2009; Martins et al., 2010). To conciliate these discrepant data, it has been hypothesized that PrP^C^ function is either dispensable or redundant with that of other proteins. Yet, recent advances, notably in the developmental biology, shed a new light on PrP^C^ functions and suggest that, perhaps, the quest for PrP^C^ functions has been made at the wrong place and/or at the wrong period of time.

**Table 1:** **Phenotypes associated to PrP invalidation/ectopic activation**.

**Life period/developmental stage**	**Type of manipulation**	**Phenotype(s)**	**Comments**	**References**
Early embryo (zebrafish)		**Lethal**		
	*PrP-1* knockdown	– gastrulation arrest due to impaired cell adhesion	– partial rescue of the morphants by the injection of PrP mRNA including mammalian sequence	Malaga-Trillo et al., 2009
Early embryo (mouse)		**Moderately severe**		
	*Prnp* knockout embryos	– transcriptomic analysis shows differential expression of genes from multiple cell pathways	– transient phenotype	Khalife et al., 2011; Passet et al., 2012
Early embryo (mouse)	PrP and Shadoo co-invalidation obtained by: *Sprn* knockdown and *Prnp* knockout	**Controversial**		
		– impaired trophoblast development, lethality	– discussed in Makhzami et al. (2014)	Young et al., 2009; Passet et al., 2012
	Double knockout	– no phenotype reported but no assessment at embryonic stage		Daude et al., 2012
Pharyngula stage (zebrafish)		**Controversial**		
	*PrP-2* knockdown	– differential expression of genes involved in multiple cell pathways	– no phenotype rescue of the morphants	Nourizadeh-Lillabadi et al., 2010
	*PrP-2* knockout	– no obvious developmental abnormalities but no transcriptomic analysis performed		Fleisch et al., 2013
Larva (zebrafish)		**Controversial**		Malaga-Trillo et al., 2009; Nourizadeh-Lillabadi et al., 2010
	*PrP-2* knockdown	– head malformations, missing neuronal structures	– no phenotype rescue of the morphants	
	*PrP-2* knockout	– no obvious abnormalities at larval stage		Fleisch et al., 2013
Late embryo/newborn (mouse)		**Minor**		
	*Prnp* knockout embryos	– increased proliferation and maturation delay of the oligodendrocyte precursor cell population in the brain	– no brain abnormalities or myelin defect	Bribian et al., 2012
		– earlier formation of dentin and enamel in the developing tooth	– no enamel defect but reduced hardness of dentin at adult stage	Zhang et al., 2011
Juvenile (mouse)		**Moderately severe**		
	*Prnp* knockout mice	– functional abnormalities and persisting cell proliferation in the cerebellum, impaired locomotor abilities	– transient phenotype	Prestori et al., 2008
Adult Brain (mouse)		**Minor**		
	*Prnp* knockout mice	– transcriptomic and proteomic analysis revealed no important differences	– no brain abnormalities or myelin defect associated	Crecelius et al., 2008; Chadi et al., 2010
					– increased protein oxidation, protein ubiquitination and lipid peroxidation		Wong et al., 2001
		– increased proliferation and maturation delay of the oligodendrocyte precursor cells		Bribian et al., 2012
		– delayed maturation of astrocytes		Arantes et al., 2009
		– decreased cell proliferation in the dentate gyrus (adult neurogenic region)		Steele et al., 2006
		– functional abnormalities in the hippocampus		Collinge et al., 1994
Adult brain (mouse)		**Minor**		
	*Prnp* overexpressing mice	– increased cell proliferation in the subventricular zone (adult neurogenic region)	– no brain abnormalities associated	Steele et al., 2006
		– shorten astrocyte maturation phase		Hartmann et al., 2013
Adult extraneural tissues (mouse)		**Minor**		
	*Prnp* knockout mice	– delayed mineralization of the continuously erupting incisors	– regeneration and renewing tissues	Zhang et al., 2011; Stella et al., 2010
				– slower regeneration of muscle fibers		
				– shortening of intestinal villi, cell cycle alterations in intestinal crypts and reduced size of desmosomes in intestinal epithelium		Morel et al., 2008
Adult (mouse)		**Minor and/or partially controversial**		Bueler et al., 1992; Collinge et al., 1994; Tobler et al., 1996; Walz et al., 1999; Nico et al., 2005; Meotti et al., 2007; Sanchez-Alavez et al., 2008; Le Pichon et al., 2009; Gadotti et al., 2012; Rial et al., 2014
	*Prnp* knockout mice	– depending of the study, no phenotype observed to minor alterations such as altered olfactory behavior	– few studies were carried out using distinct genetic background	
Adult (zebrafish)		**Minor**		Fleisch et al., 2013
	*PrP-2* knockout	– increased susceptibility to a convulsant drug	
		– kinetics alteration of NMDA receptors	
Adult (zebrafish)	*PrP-1* knockout	**Unknown**	– no transgenic (inducible) line established	
Aged animal (mouse)		**Minor**		Rial et al., 2009; Massimino et al., 2013
	*Prnp* knockout mice	– behavior alterations	
Aged animal nerves (mouse)		**Minor**		
	*Prnp* knockout and conditional *Prnp* knockout	– increased myelin abnormalities in peripheral nerves	– several genetic background analyzed	Bremer et al., 2010

## Early developmental expression of the PrP genes in selected vertebrates

The apparent lack of a phenotype in mice invalidated for PrP^C^ sounds at odds with the very early and quasi-ubiquitous expression pattern of the protein at embryonic and postnatal stages, respectively. Expression of the gene encoding PrP—*Prnp*—is detected in post-implantation embryo from embryonic day (E) 6.5 in extra-embryonic regions (Manson et al., 1992) and from E7.5 (late allantoic bud stage) in cardiac mesoderm (Hidaka et al., 2010). *Prnp* expression is then observed in the developing central nervous system and heart around E8 before extending rapidly almost to the entire embryo (Tremblay et al., 2007). *Prnp* may even be expressed earlier on as PrP mRNAs have been found maternally expressed in the zygotes of *Xenopus laevis* and zebrafish (*Danio rerio*) (van Rosmalen et al., 2006; Malaga-Trillo et al., 2009).

Two PrP homologs, PrP-1, PrP-2 and a more divergent form, PrP-3, have been identified in zebrafish (Cotto et al., 2005; Rivera-Milla et al., 2006). The expression patterns of the corresponding genes (*PrP-1*. *PrP-2*, and *PrP-3*, respectively) in zebrafish embryos are partially conflicting (Cotto et al., 2005; Malaga-Trillo et al., 2009) although both studies agree on their spatio-temporal complementarity. Both studies describe early PrP gene expression from the mid-blastula stage although it is *PrP-2* according to the Cotto et al. study and *PrP-1* according to Malaga-Trillo et al. At pharyngula stage (24–48 h postfertilization), both studies show a strong and broad expression of *PrP-2* in the developing central nervous system. Additionally, the Cotto et al. study reports the expression of *PrP-1* in the floor plate, an essential organizing center of the central nervous system and in the peripheral nervous system around the same developmental period. The expression of *PrP-2* and *PrP-3* in the developing neuromasts (sensory organs) is also described at the larval stage. PrP-encoding genes are also expressed in developing organs or tissues outside the nervous system such as the heart (*PrP-2* and *PrP-3*), the kidney (*PrP-2*), the pectoral fins (*PrP-2* and *PrP-3*) and the intestinal epithelium (*PrP-2*) (Cotto et al., 2005).

## The embryo spills the beans

In sharp contrast with the situation in mammals, knockdown (KD) of *PrP-1* or *PrP-2* by morpholino injection led to severe biological phenotypes in zebrafish (see Table 1): gastrulation arrest for *PrP-1* KD (Malaga-Trillo et al., 2009) and malformations of the brain and the eyes associated to a reduced number of peripheral neurons for *PrP-2* KD (Malaga-Trillo et al., 2009; Nourizadeh-Lillabadi et al., 2010). However, *PrP-2* KD-induced phenotype is subject to caution as *PrP-2* knockout (KO) by zinc finger nuclease-induced targeted mutation did not lead to any obvious developmental phenotype (Fleisch et al., 2013). Additional and aspecific effects due to morpholino injection may have occurred. There is less concern about *PrP-1* KD phenotype as partial rescue was obtained by the injection of *PrP-1* mRNA, *PrP-2* mRNA and, remarkably, mouse *Prnp* mRNA (Malaga-Trillo et al., 2009), strongly suggesting that zebrafish and mammalian PrPs may share conserved functions.

PrP inactivation studies in zebrafish allow investigating PrP^C^ functions. They also favor a more critical role of PrP^C^ in early development rather than during postnatal stages (see Table 1). Accordingly, transcriptomic analyses (by RNAseq) of *Prnp^−/−^* versus wild-type mouse embryos identified numerous genes differentially expressed (73 and 263 genes at E6.5 and E7.5, respectively) (Khalife et al., 2011), while only 1 gene was found differentially expressed in adult brains (Chadi et al., 2010). Proteomic analysis also failed to identify major alterations (Crecelius et al., 2008) although many cellular stress markers such as protein oxidation, protein ubiquitination, and lipid peroxidation were reportedly activated in the brains of *Prnp^−/−^* versus wild-type mice (Wong et al., 2001). This suggests either that PrP^C^ is quite dispensable in the adult brain or that, in a *Prnp^−/−^* context, alternative mechanisms are activated during the brain development to compensate the absence of PrP^C^. Supporting the second hypothesis, the number of genes differentially expressed in the adult *Prnp^−/−^* mouse brain was substantially higher upon postnatal depletion in neurons than embryonically (Chadi et al., 2010), albeit to a less extent than in the embryo. The genes differentially expressed in the embryo covered various cell functions including adhesion, apoptosis and proliferation and are related to heart formation and blood vessel development, vascular diseases, immune response, nervous system development, and prion diseases (Khalife et al., 2011). Interestingly and in line with the idea of evolutionary conserved functions of PrPs, similar biological functions were impacted by *PrP-2* KD in zebrafish, as revealed by transcriptomic analyses: development including that of the cardiovascular and nervous systems specifically, cell death, cellular assembly, cell cycle, immunological, and neurological diseases (Nourizadeh-Lillabadi et al., 2010).

Finally, the KD of the *Sprn* gene encoding a PrP-related protein, Shadoo resulted in embryonic lethality between E8 and E11 specifically in *Prnp^−/−^* embryos, supporting a role for PrP^C^ and Shadoo in mouse embryogenesis, notably cell adhesion and placenta development, and a possible functional redundancy between the two proteins (Young et al., 2009; Passet et al., 2012). However, the recent generation of viable *Prnp^−/−^; Sprn^−/−^* animals is challenging this view (Daude et al., 2012). These apparently conflicting observations are discussed in Makhzami et al. (2014).

Thus, studying the consequences of PrP inactivation during developmental stages rather than in adult animals could be a more efficient, although not easier, strategy to decipher PrP^C^ functions. Additionally, studying PrP^C^ implications in regenerative processes and in renewing tissues could also be an informative approach (see Table 1).

## The current limits of *in vivo* studies in mammals

As PrP^C^ is mediating neuronal dysfunction and degeneration in prion diseases and reportedly in Alzheimer's disease (Lauren et al., 2009; Klyubin et al., 2014), it has been logically suspected to play a role in neuronal homeostasis and during neural development. As PrP^C^ functions in mature neurons are not under the scope of the present review, we will not discuss them and only focus on developmental aspects. *In vivo* direct evidence supporting a role of PrP^C^ in neural development is quite elusive and PrP^C^ expression pattern remains one of the strongest pro arguments. Indeed, PrP^C^ expression increases as neurons mature, although it is not detected in mitotically active neural progenitor cells (Steele et al., 2006; Peralta et al., 2011). Other experimental evidence was obtained from the comparative study of WT and *Prnp^−/−^* and/or *Prnp* overexpressing animals (see Table 1). The levels of PrP^C^ were found to positively regulate the proliferation in neurogenic regions in adult mice (Steele et al., 2006; Bribian et al., 2012) and the maturation of astrocytes in the developing brain (Arantes et al., 2009; Hartmann et al., 2013). Conversely, the absence of PrP^C^ was associated with an increased proliferation in oligodendrocyte precursor cells both in embryos and adults (Bribian et al., 2012). However, no brain morphology abnormalities or myelin defects could be observed in *Prnp^−/−^* animals, casting doubt on a crucial role of PrP^C^ in cell proliferation and maturation during brain development or, at least, suggesting the occurrence of compensatory mechanisms. Yet, electrophysiological recordings on hippocampus from *Prnp^−/−^* mice (Collinge et al., 1994) and on cerebellum from juvenile *Prnp^−/−^* mice (Prestori et al., 2008) revealed functional alterations. However, no hippocampus-related behavioral or learning alterations were observed in *Prnp^−/−^* mice (Bueler et al., 1992) suggesting the hippocampus functional alterations are minor or at least compensated. Conversely, cerebellum functional alterations were found associated to a failure of juvenile *Prnp^−/−^* mice in motor control test and to protracted cell proliferation in the cerebellum granular layer at the third week of postnatal development although all these abnormalities vanished in older animals (Prestori et al., 2008). Taken together, these arguments suggest a delay in the maturation of the cerebellar granule cells in *Prnp^−/−^* mice.

Further dissecting the underlying mechanisms responsible for the subtle phenotypes in *Prnp^−/−^* mice is a particularly arduous task *in vivo*. For example, determining whether the effects are cell-autonomous would require the use of several transgenic lines or genetic chimeras to limit the expression of PrP^C^ to a subset of cells. And this question is far from being irrelevant as PrP^C^ is a cell surface protein. Such topology could elicit specific cell responses in neighboring cells. Additionally, PrP^C^ may act at distance since secreted forms of have been described both *in vitro* and *in vivo* (Borchelt et al., 1993; Harris et al., 1993).

## Contributions of *in vitro* and *ex vivo* models to understanding the role of PrP^C^ in neural development

The use of *in vitro* or *ex vivo* models has generated a wealth of data with regard to the involvement of PrP^C^ in neural development and, importantly, has comforted the putative functions suggested by the *in vivo* approaches. Moreover they often allow pinpointing more precisely the cellular processes and signaling pathways involving PrP^C^. Collectively, these experimental data argue that PrP^C^ positively regulates (1) the differentiation of pluripotent progenitors cells toward the neural lineage (Peralta et al., 2011), (2) the proliferation of neural progenitor cells (Prodromidou et al., 2014) (3) the self-renewal of neural progenitor cells (Santos et al., 2011; Prodromidou et al., 2014) (4) the neuronal differentiation in terms of choice of cell fate (Steele et al., 2006) and (5) acquisition of neuronal features (Graner et al., 2000). However, how PrP^C^ fulfills these different actions is only partially understood. The current idea is that PrP^C^ is part of multiprotein signaling complexes, able to bind various partners, and participate to signal transduction events along different cellular pathways depending on the cellular context (Caughey and Baron, 2006; Watts et al., 2009; Stuermer, 2011; Hirsch et al., 2014). Non-exhaustively, the interaction between PrP^C^ and one of its identified ligand, Stress inducible protein 1 (STI1), has been shown to promote the self-renewal of the neural progenitor cells (Santos et al., 2011). Besides, interaction with the neural cell adhesion molecule (NCAM) can induce their neuronal differentiation (Prodromidou et al., 2014). In cultured neurons, STI1 binding to PrP^C^ increases protein synthesis (Roffe et al., 2010), STI1 and Laminin-γ1 binding promotes intracellular Ca^2+^ increase and axonogenesis (Santos et al., 2013), and vitronectin binding, axonal growth (Hajj et al., 2007). Experimental evidence also favors a role of PrP^C^ in glia development since the lack of PrP^C^ promotes the proliferation of oligodendrocyte precursor cells and delays their differentiation in culture (Bribian et al., 2012). Studies in astrocyte primary cultures revealed that the involvement of PrP^C^ in astrocytes maturation is dependent upon its interaction with STI1 and the downstream activation of ERK1/2 (Arantes et al., 2009). Of note, PrP^C^ action does not necessarily rely on PrP^C^ expression solely in the cell population considered. For instance, proper neuritogenesis has been shown to require PrP^C^ expression in both neurons and surrounding astrocytes in co-cultures experiments (Lima et al., 2007). Beyond a role in the interactions between both populations, PrP^C^ serves as a neuronal receptor for laminin (Graner et al., 2000) and is also involved in the organization of the extracellular matrix by the astrocytes, especially through laminin deposition (Lima et al., 2007).

It should also be stressed that PrP^C^ partners and the cellular context could tightly determine the final consequences of PrP^C^ stimulation. For example PrP^C^ interaction with contactin-associated protein (Caspr) protects Caspr from proteolysis allowing it to inhibit more efficiently neurite outgrowth (Devanathan et al., 2010). On the opposite, PrP^C^ and NCAM interactions allow stabilization of NCAM in lipid rafts allowing Fyn kinase activation and promoting neurite outgrowth (Santuccione et al., 2005).

*In vitro*, PrP^C^ is thereby found involved in all the major steps of neural development. Since it notably regulates the self-renewal and differentiation processes in neural progenitor cells, the question arises as to whether this role can be extended to other cell types.

## What the study of stem cells reveals

The role of PrP^C^ is far from being restricted to neural development and a growing body of evidence support its involvement in stem cell biology see also Martin-Lannerée et al. (2014). Whether PrP^C^ is expressed in embryonic stem cells (ESC) at basal state remains unclear (Lee and Baskakov, 2010; Miranda et al., 2011), possibly due to limitations in the sensitivity of the detection methods, choice of the target (i.e., the gene or the protein itself) and methods of production and culture of the cells. Nevertheless, there is a consensus that PrP^C^ expression progressively increases during stem cell differentiation (Miranda et al., 2011; Lee and Baskakov, 2013). To understand the role played by PrP^C^ during early embryogenesis, the consequences of *Prnp* KD or overexpression were studied in human ESC (Lee and Baskakov, 2013). Forcing PrP^C^ expression under self-renewal conditions was found to alter cell cycle regulation and to promote ESC differentiation. Blocking PrP^C^ expression in differentiating ESC also impacts on cell cycle regulation and inhibits the differentiation toward ectodermal lineages, in line with PrP^C^ functions in neural differentiation (Peralta et al., 2011) and the reduced expression of *Nestin*, an ectodermal marker, observed in ESC in the absence of PrP^C^ (Miranda et al., 2011; Peralta et al., 2011). Mesodermal and endodermal differentiations were, however, not affected. Finally, overexpressing PrP^C^ in differentiating ESC promotes proliferation and inhibits differentiation toward all the lineages (Lee and Baskakov, 2013).

Further supporting PrP^C^ function in stem cell biology, PrP^C^ is expressed in hematopoietic stem cells and promotes their self-renewal (Zhang et al., 2006). PrP^C^ is therefore involved in the self-renewal and differentiation of stem cells although its role and requirement seem to vary along the differentiation process and the cell lineage considered. Identifying the molecular mechanisms at play is a particularly challenging task since self-renewal, differentiation and proliferation are highly intertwined processes impacting each other.

## The role of PrP^C^ in developing and renewing tissues outside the nervous system

Looking outside the central nervous system in newborn to full-grown animals allowed pinpointing subtle phenotypes in the absence of PrP^C^ (see Table 1), in perfect consistency with PrP^C^ widespread expression. PrP^C^ is expressed in developing human and murine teeth and study of dental cell cultures and teeth from *Prnp^−/−^* animals revealed multiple alterations during tooth formation (Schneider et al., 2007; Zhang et al., 2011). *In vitro*, embryonic dental mesenchymal cells from *Prnp^−/−^* embryos proliferate more rapidly and differentiate earlier and coherently, *Prnp^−/−^* newborn mice exhibit earlier formation of mesenchymally- and epithelially-derived dentin and enamel, respectively (Zhang et al., 2011).

PrP^C^ is also abundantly expressed in muscles and, although no abnormalities could be observed in a physiological context, it is involved in the regeneration of skeletal muscles in adult mice. Regeneration of locally damaged muscle fibers occurs at slower pace in *Prnp^−/−^* animals. Yet total recovery is finally achieved (Stella et al., 2010). This is associated with a longer phase of proliferation and a delayed maturation of muscle precursors, likely due to a reduced level of released myogenic factors (Stella et al., 2010). However, the identity of the releasing cells is difficult to establish, showing again the limits of the *in vivo* paradigm.

Another extra-neural tissue where PrP^C^ depletion induces alterations is the intestinal epithelium, a constantly renewing tissue. PrP^C^ is expressed by human enterocytes (Morel et al., 2004) and *Prnp^−/−^* animals exhibit shorter intestinal villi associated to an increased number of mitotic cells (Morel et al., 2008).

Collectively, these observations support a role of PrP^C^ in the regulation of cell cycle and cell differentiation extraneurally. But how can these functions be exerted?

## PrP^C^ regulates cellular adhesion, extra-cellular matrix interactions, and cytoskeleton remodeling

*PrP-1* KD-induced gastrulation arrest in zebrafish embryos is due to impaired morphogenetic cell movements. This defect is, at least partially, caused by the disruption of cellular adhesion (Malaga-Trillo et al., 2009). The study revealed that PrP-1 normally accumulates at cell-cell contacts at early embryonic stages and regulates cell adhesion, including E-cadherin processing and/or storage, in a cell-autonomous way. Such functions may be conserved in mammals as injection of mouse mRNA *Prnp* partially rescued the *PrP-1* KD-induced phenotype (Malaga-Trillo et al., 2009). Consistent with this idea, a number of experimental evidence have linked PrP^C^ to cell adhesion and intimately associated processes such as cytoskeleton remodeling and interactions with extra-cellular matrix (Petit et al., 2013): (i) the expression pattern of PrP^C^. PrP^C^ is expressed at the cell-to-cell contacts, growth cone, and other extending process tips in cultured neural progenitors and neurons (Santuccione et al., 2005; Devanathan et al., 2010; Miyazawa et al., 2010), cell-to-cell junctions in human intestinal epithelium villi (Morel et al., 2004), cell-to-cell contacts in primary cultured endothelial cells (Viegas et al., 2006), focal adhesions in HeLa cell lines (Schrock et al., 2009) and the apical face of ameloblasts of developing teeth (Zhang et al., 2011); (ii) relevant PrP^C^ partners and ligands, such as cell junction proteins including integrins, the cytoskeleton protein actin and components of the extra-cellular matrix (Graner et al., 2000; Schmitt-Ulms et al., 2001; Morel et al., 2004, 2008; Nieznanski et al., 2005; Hajj et al., 2007; Watts et al., 2009); (iii) alterations in cell adhesion, cytoskeleton dynamics, and extra-cellular matrix interactions upon ectopic expression or deletion of PrP^C^ in different cell systems. *Prnp* KD leads to alterations in the actin cytoskeleton, remodeling of focal adhesions and over-production of fibronectin in neuronal progenitors (Loubet et al., 2012), to disruption of adherens junctions in carcinoma cells (Solis et al., 2012) and to miss-localization of cell junction-related proteins in enterocytes (Morel et al., 2008). As for *Prnp* overexpression, it promotes aggregation and filopodia formation as well as alterations of the focal adhesion dynamics in neuroblastoma N2a cells (Mange et al., 2002; Schrock et al., 2009). Subtle changes were even noticed *in vivo* such as a decrease in the length of desmosomes in the intestinal epithelium of *Prnp^−/−^* mice (Morel et al., 2008).

Interestingly, the alterations induced by the deletion of PrP^C^ can be partially rescued by neutralizing antibodies to specific integrins: use of β 1 and β 5 integrins was found to restore neuritogenesis in differentiating PrP-deficient neuronal progenitors (Loubet et al., 2012) and *Nanog* expression in differentiating PrP-deficient ESC (Miranda et al., 2011), respectively. Moreover, modulation of integrin activity was suspected to be a spontaneous compensatory mechanism occurring in the absence of PrP^C^ (Hajj et al., 2007). This last observation supports an involvement of PrP^C^ in extra-cellular matrix interactions and suggests a possible adaptive mechanism to the absence of PrP^C^, which may account for the lack of drastic phenotypes in *Prnp^−/−^* animals.

Taken together, these observations demonstrate a clear involvement of PrP^C^ in the regulation of cell adhesion, the interactions with the extra-cellular matrix and the cytoskeleton dynamics. The links between these functions and the role of PrP^C^ in the regulation of cell proliferation and differentiation have been the focus of few studies (Miranda et al., 2011; Loubet et al., 2012). Are they under-estimated? And are these functions still ongoing in adult organism?

## Studying PrP^C^ functions during aging

Little attention has been devoted to the potential functions of PrP^C^ during aging (see Table 1 and also Gasperini and Legname, 2014). It is commonly accepted that the cellular machinery deteriorates or, at least, evolves with aging. As the potential development of adaptive mechanisms to the absence of PrP^C^ is assumed to complicate the study of PrP^C^ functions in *Prnp^−/−^* mice, the study of aged animals could be a way to bypass such difficulty: because of the age-related alterations accumulation, the tolerance to PrP^C^ depletion could partially break. In line with this scenario, behavioral differences between WT and *Prnp^−/−^* mice seem more pronounced when performed on aged animals (Rial et al., 2009; Massimino et al., 2013). Age-related physiological traits can also be found amplified in absence of PrP^C^. Myelin abnormalities in the peripheral nervous system accumulate with aging (Verdu et al., 2000) and sciatic nerves from moderately aged mice devoid of PrP^C^ (in selected or all cell populations) showed highly precocious and increased demyelination in the absence of neuronal PrP^C^ (Bremer et al., 2010). If the axons-glia interactions are known to play a key role in myelination during development (Sherman and Brophy, 2005), their role in myelin maintenance in adulthood is less well-understood. However, myelin maintenance is clearly established as an active process (Bremer et al., 2011). The early demyelination observed in the absence of neuronal PrP^C^ suggests axonal PrP^C^ participates in myelin maintenance and is likely required, directly or indirectly, in axons-Schwann cells communication.

Thus, aging may perhaps be the ultimate stage to unravel the mystery of PrP^C^ function(s). However, it has to be considered that aging could also impact on PrP^C^ and/or its function(s). For instance, the age-related change in the cholesterol/sphingolipids ratio in lipid rafts is suspected to alter PrP^C^ compartmentalization (Agostini et al., 2013), which could ultimately impact on its functions. A functional study of PrP^C^ during aging could therefore be difficult to analyse due to the numerous changes occurring at the same time.

## Concluding remarks

A major function of PrP^C^, consistent with its cell surface expression consists in the modulation of signaling pathways in response to various cues. The panel of putative PrP^C^ ligands is quite large, including soluble ligands, extra-cellular matrix components, and adhesion molecules. Their modes of interaction with PrP^C^ are variable, including cooperation (Santos et al., 2013) and both cis- and trans-interactions for a same ligand (Santuccione et al., 2005). The cellular context can strongly influence the composition of the PrP^C^-multiprotein complexes and the sub-cellular localization of PrP^C^ is highly dynamic: it can cycle rapidly from the plasma membrane to internal membranes (Griffiths et al., 2007) possibly in response to a given stimulus (Lee et al., 2001; Brown and Harris, 2003; Caetano et al., 2008). All these elements taken together may allow a specific response to a given signal with regard to the cell type and differentiation state. This may explain why PrP^C^ appears to be involved in a multitude of functions. Regarding development, this view perfectly fits the requirement of different stem and progenitor cells by eliciting appropriate cell responses to adapt efficiently to different microenvironments. But how PrP^C^ precisely impacts on stemness maintenance and differentiation processes remains unresolved. The potential implication of PrP^C^ in cell polarization and cytoskeleton remodeling in differentiating stem cells is a very attractive hypothesis and should obviously deserve further investigation. Cell polarity and cytoskeleton dynamics are life-long important cell processes, notably during cell fate determination. They have been studied more particularly during drosophilia neurogenesis and in mammalian neuroepithelial cells (Fietz and Huttner, 2011), but they are fundamental processes for all sort of stem and progenitor cells such as hematopoietic progenitor cells (Bullock et al., 2007). Our unpublished data (Halliez et al., in preparation) reveal a polarized expression of PrP^C^ in neural and cardiovascular progenitor cells of the developing mouse embryo and therefore are compatible with such idea.

If PrP^C^ is involved in so many cell lineages, understanding the lack of drastic phenotype in PrP^C^-depleted animals remains an ongoing issue. Yet, a modulation of the cell maturation phase (generally delayed but also shortened) is consistently observed in different models (*Prnp* KO but also KD and overexpression) and may therefore be the general price to pay for the loss of PrP^C^ (see Table 1). This may have a more selective impact on free-range than on laboratory animals (for example acquisition of perfect motor control) and justify the high degree of PrP conservation through evolution. Moreover, building an organism is a highly complex task requiring spatial and temporal fine-tuning of a broad number of biological processes via multiple regulatory networks. Cells, tissues and organs do not develop separately along each other but rely on multiple interactions to develop in a concerted, interconnected, and time-scheduled manner. Partial redundancies into and between signaling pathways ensure robustness to the system. That two or more proteins may exert overlapping functions during development and therefore allow tolerance to the loss of one of them is not unprecedented (Yoon et al., 2005; Santamaria and Ortega, 2006; Nicolae et al., 2007). Moreover, it is not unheard-of, notably in plants where environment adaptation is largely studied, that redundant genes in a regular context allow survival under environmental modifications and stress (Wu et al., 2002). Interestingly, *PrP* KO animals seem to react differentially than WT animals to stressful situations (Nico et al., 2005) and to alcohol (Rial et al., 2014) and show increased susceptibility to convulsant agents (Walz et al., 1999; Fleisch et al., 2013) (see Table 1). The general function of PrP^C^ could be to facilitate responses to external stimulus/factors at the cell- and at the organism-scale. Yet, laboratory animals are raised in standardized and protective environment and are often homogenous genetically. In such conditions, the time window to apprehend the cell specific functions of PrP^C^ may be restricted to the developmental and perinatal phases, with so much information provided and so much transformation occurring at the same time.

### Conflict of interest statement

The authors declare that the research was conducted in the absence of any commercial or financial relationships that could be construed as a potential conflict of interest.
